# Phytochemical composition analysis and evaluation of *in vitro* medicinal properties and cytotoxicity of five wild weeds: A comparative study

**DOI:** 10.12688/f1000research.22966.1

**Published:** 2020-06-02

**Authors:** Pranabesh Ghosh, Chandrima Das, Swagata Biswas, Sudip Kumar Nag, Alolika Dutta, Maitrayee Biswas, Sayantan Sil, Labani Hazra, Chandreyi Ghosh, Shaktijit Das, Moumita Saha, Nasim Mondal, Suprodip Mandal, Anirban Ghosh, Srabani Karmakar, Sirshendu Chatterjee

**Affiliations:** 1 Department of Biotechnology, Techno India University, West Bengal, EM-4, Salt Lake, Kolkata, West Bengal, 700091, India; 2 School of Pharmacy, Techno India University, West Bengal, EM-4, Salt Lake, Sector- V, Kolkata, West Bengal, 700091, India; 3 Department of Zoology and Immunobiology Laboratory, Panihati Mahavidyalaya, Sodepur, Kolkata, West Bengal, India

**Keywords:** Heliotropium indicum, Tridax procumbens, Cleome rutidosperma, Commelina benghalensis, Euphorbia hirta, HPLC, Antioxidants.

## Abstract

**Background: **Medicinal plants are a source of phytochemicals and they are used for the treatment of several oxidative stress-related or other diseases for their effectiveness, low toxicity and easy availability. Five traditionally used and less characterized herbaceous weeds of West Bengal, India, namely, *Heliotropium indicum*, *Tridax procumbens*, *Cleome rutidosperma*, *Commelina benghalensis* and *Euphorbia hirta*, ****were investigated for the current research study.

**Methods:** Aqueous and 70% ethanolic extracts of the leaves were analyzed for estimation of essential phytochemicals and to evaluate their *in vitro* antioxidant status, medicinal properties and cytotoxic effects. To the best of our knowledge, several assays and comparative evaluations using these herbs are reported for the first time. For quantitative study, UV-vis spectrophotometry and high-performance liquid chromatography with diode array detector HPLC-DAD techniques were used. Antibacterial properties were investigated using the Kirby-Bauer disc diffusion method. For *in vitro* anti-lithiatic study, a titration method was used. The cell viability assay was done using peripheral blood mononuclear cells.

**Results:** The aqueous extract exhibits higher content of polyphenols, flavonoids, tannins and inhibition percentage values for free radical scavenging assays, whereas the 70% ethanolic extract exhibits higher content of alkaloids and cardiac glycosides. HPLC-DAD analysis of 70% ethanolic extracts led us to identify 10 predominant phenolic constituents. *Euphorbia hirta* extracts showed minimum cytotoxicity (cell death *~2.5% and 4* *%* in water and 70% ethanolic extract, respectively *)*, whereas *Cleome rutidosperma* and *Tridax procumbens’* 70% ethanolic extracts showed higher cell death (~13% and 28%, respectively), compared with the control (cell death ~10-12%).

**Conclusions:** The study concluded that of all the medicinal weeds selected for the current study, *Euphorbia hirta *possesses the highest amount of bioactive compounds and hence exhibits the highest *in vitro* antioxidant activity and promising *in vitro* medicinal properties.

## Introduction

At present, many people are cautious about synthetic drugs usage because of their side effects and high price. On the contrary, herbal medicines have been continuously used in healthcare treatment system due to their cost effectiveness, better feasibility in the human physiological system and with minimal side effects. India is a vast source of medicinal and aromatic plants. Among its diverse and rich collection of medicinal plants, we have chosen five wild ethnomedicinal weeds and those are very less characterized and available with reported various bioactivities ^1– 4^.

These weeds grow in similar habitats, though they belong to different families. Botanically, one species of plant can considered as a valuable crop or medicinal plant, while another species in the same genus can be treated as a dangerous weed. Many plants that are widely accepted as weeds are also grown in gardens and other cultivated areas; these are known as beneficial weeds ^1– 3, 5– 9^. The current study focuses on searching for those medicinal weeds which have enormous future prospects for pharmaceutical industries.

*Heliotropium indicum* Linn. (Boraginaceae) is an annual herbaceous weed commonly known as the Indian heliotrope and is native to India ^5^. *Tridax procumbens* Linn. (Asteraceae) is an annual herbaceous weed and commonly known as coat buttons; it is native to tropical America ^6^. *Cleome rutidosperma* DC. (Cleomaceae) is an annual herb commonly known as Fringed Spider Flower; it is native to tropical Africa ^7^. *Commelina benghalensis* Linn. (Commelinaceae) is an annual herb native to tropical Asia and Africa, commonly known as Bengal dayflower ^8^. *Euphorbia hirta* Linn. (Euphorbiaceae) is an annual herb commonly known as asthma plant, and is native to India and Central America ^9^.

Leaf, stem, root or sometime the complete plant possess the property to scavenge reactive oxygen species (ROS) or reactive nitrogen species (RNS), the main causative agents of oxidative stress and cellular damage which results in numerous diseases and disorders ^10, 11^. ROS or RNS includes a variety of free radicals such as superoxide anion (O _2_^-^), hydroxyl radical ( ^.^OH), nitric oxide radical (NO ^.^), and peroxyl radicals (ROO ^.^), and non-free radical species like hydrogen peroxide (H _2_O _2_), ozone (O _3_), hypochlorous acid (HOCl), nitrous acid (HNO _2_) ^12^. The primary site of ROS production in the cell is the mitochondrial respiratory chain. Natural antioxidants protect us from oxidative stress by reducing the free radicals to nontoxic products. However, healthy cells have their own natural ROS or RNS defense mechanisms which actively eliminate free radicals through enzyme-mediated systems like superoxide dismutases, peroxidases, catalases, and glutathione peroxidases ^10– 12^.

Life-threatening microbial infections brought on by various pathogens are a significant cause of morbidity and mortality within immune-compromised individuals. Plant-derived natural antioxidants can play a vital role to protect from those microbial infections ^13– 15^. Administration of α-amylase inhibitors are necessary to keep diabetic patient glucose levels under control ^16^. Determination of *in vitro* anti-diabetic and free radical scavenging activities are vital because it prevents both microvascular and macrovascular complications of diabetes. Medicinal plants are the rich source of α-amylase inhibitors and can be utilized for prevention or therapeutic measures ^17, 18^.

Plant derived antioxidants have profound activities in inflammatory disorders. Free radicals are essential mediators that provoke or sustain inflammatory or autoimmune diseases like rheumatoid arthritis, and consequently, their neutralization by antioxidants can give relief from inflammation and swelling ^19– 21^.

Urolithiasis is a disorder of the urinary tract, which may happen due to the oxidative stress by free radical generations ^22– 24^ and hence natural free radical scavengers are the best answer for that disease as well. Although medicinal plants have many bioactivities, sometimes their use should be prohibited due to their toxic effects; therefore, determination of cytotoxic effects of the medicinal plants’ extracts is a pre-requisite for their ethnomedicinal use against various physiological disorders of human beings ^25^.

The therapeutic potential of the wild plants can be determined by measuring their phytochemical constituents, *in vitro* medicinal properties and toxicological effect. Therefore, the need arises to estimate and analyze the phytochemical constituents of these five wild medicinal plants leaves which are basically responsible for curing several physiological disorders and complications. The current research investigation aims to quantify the phytochemical constituents, and to evaluate the *in vitro* antioxidant activities and other important *in vitro* medicinal properties of the medicinal weeds under study. Next, cytotoxicity of the leaf extracts is to be determined using a cell viability assay and compared. To the best of our knowledge, the comparative evaluations of these medicinal herbs are reported for the first time.

## Methods

### Collection, identification and extraction of plant material

Fresh leaves of the five medicinal plants were collected from Salt Lake City, Kolkata, West Bengal and India and authenticated by Botanical Survey of India, Central National Herbarium, Howrah, West Bengal, and India. The fresh leaves of the herbs were washed with clean water and dried at an ambient temperature for 30 days under shade. Dried leaves were made powdered and extracted using double-distilled water or 70% ethanol (1 g of powdered leaf was extracted with 50 ml solvent). The solution of each extract was then clarified using filter paper and stored at 4°C. The extracts were appropriately diluted for during further studies.

### Collection of microbial strains

A total of four Gram-positive ( *Bacillus subtilis*, *Bacillus cereus*, *Staphylococcus aureus*, and *Staphylococcus epidermidis*) and four Gram-negative ( *Escherichia coli*, *Vibrio cholera*, *Pseudomonas aeruginosa*, and *Klebsiella pneumonia*) bacterial strains were used to measure the antimicrobial activity. The bacterial strains were obtained from the Department of Microbiology, Calcutta University, West Bengal and India.

### Chemicals and reagents

All the chemicals and reagents used in the experiments were of analytical (AR) grade and purchased from Institutional enlisted chemical suppliers. For quantitative assays, Systronics 117 model spectrophotometer was used to determine the specific optical density.

### Phytochemical analysis

***Estimation of total polyphenol content.*** Quantitative estimation of the total phenolic compounds was done in triplicate by using the Folin-Ciocalteu (Merck, India) method of Singleton *et al*. ^26^ with slight modifications. In brief, 1500 µl of diluted FC reagent was mixed with 300 µL of leaf extract thoroughly. Next, 1200 µl 7.5% Na _2_CO _3_ was added to it, vortexed and the total reaction mixture (3 mL) was incubated at dark for 2 h. The absorbance was measured at 765 nm. Gallic acid (SD Fine-Chem, India) was used as standard. The total phenolics contents were expressed in mg gallic acid equivalent/g dry weight.

***Estimation of total flavonoid content.*** Total flavonoids content was quantified in triplicate by the aluminium chloride (Merck, India) colourimetric assay according to standard protocol of Zhishen *et al*. ^27^ Quercetin (SRL, India) was used as standard. The absorbance was read at 510 nm. The total flavonoids content was expressed in mg quercetin equivalent/g dry weight.

***Estimation of total tannin content.*** The total tannins content was evaluated by using Broadhurst and Jones ^28^ method in triplicate. Tannic acid (SRL-92101, India) was used as a standard reagent. The absorbance was measured at 500 nm. Total tannins content was expressed in mg tannic acid equivalent/g dry weight.

***Estimation of total alkaloid content.*** Total alkaloids content was determined by using the method of Fazel *et al.* ^29^ in triplicate. The absorbance was measured at 470 nm. Caffeine (SRL, India) was used as standard. Total alkaloid content was expressed in mg caffeine equivalent/g dry weight.

***Estimation of total cardiac glycosides content.*** Total cardiac glycosides content was estimated according to the method of Solich *et al.* ^30^ in triplicate. Digoxin (SRL, India) was used as standard. Absorbance was read at 495 nm. The total cardiac glycoside was expressed in mg digoxin equivalent/g dry weight.

***Estimation of total saponin content.*** Total saponin content was quantified using a standard method (20% ethanol) in triplicate ^31, 32^. The saponin content was calculated by using the following equation:

Totalsaponincontent(%)=(WEP/WPS)×100

Where WEP = weight of dried end product, WPS = weight of powdered sample (initial).

***High-performance liquid chromatography (HPLC) profiling of the 70% ethanolic extracts.*** The HPLC profiling of ten important bioactive phenolic acids, gallic acid (GA), catechin hydrate (CH), chlorogenic acid (CHA), caffeic acid (CA), syringic acid (SYA), p coumaric acid (pCA), sinapic acid (SIA), coumarin (CM), quercetin (QE) and kaempferol (KMP), was carried out using an Agilent Technologies 1260 Infinity liquid chromatography. Standard reagents were purchased from Sigma-Aldrich (USA). The peak area was calculated by Open Lab CDS version 2.0 software. In the experiments the polyphenolic compounds were separated under the following conditions: Phenomenex-C18 (2)-column (250 mm×4.6 mm i.d.; Luna 5-μm particle diameter 100 Å), the diode array detector (DAD) was set at 280 nm; the mobile phase consisted of 3% acetic acid water and acetonitrile (Himedia, India). Prior to use, the solutions were degassed in an ultrasonic bath and filtered through 0.22-μm membranes. The flow rate was 0.9 ml/min in gradient conditions. The injection volume was 20 μl. All the separations are carried out at ambient temperature. The gradient elution of solvent A (water-acetic acid) and solvent B (acetonitrile) had a significant effect on the resolution of compounds according to the procedure: 0 min, 100% (A); 5 min, 95% (A); 17 min, 85% (A); 40 min, 60% (A); 60 min, 50% (A); 65 min, 50% (A); 70 min, 100% (A) ^33, 34^.

### Evaluation of antioxidant activity

***ABTS free radical scavenging capacity assay.*** ABTS (2, 2-azino-bis-3-ethylbenzthiazoline-6-sulphonic acid, Tokyo Chemical Industry, Japan) radical cation decolourization assays were carried out in triplicate to determine the stable free radical scavenging property of extracts by the help of the standard method of Re *et al*. ^35^ with slight modifications. In brief, 5 µL of leaf extract was mixed with 2.995 µl of ABTS reagent thoroughly, vortexed and the total reaction mixture (3 mL) was incubated at dark for 30 min. Next, absorbance was read at 734 nm. Ascorbic acid (Merck, India) was used as a standard. ABTS stable free radical scavenging property was expressed in ascorbic acid equivalent as well as the inhibition percentages were determined using the following equation: ^35– 37^

ABTSinhibition(%)=(ControlOD−SampleOD)/ControlOD×100

***DPPH free radical scavenging capacity assay.*** The DPPH (1, 1-diphenyl-2-picrylhydrazyl, SRL, India) stable free radical scavenging property of the extracts was carried out by the help of using the standard method of Shen *et al*. (2010) with slight modifications. In brief, 0.1 mM DPPH solution was made in methanol and 50 µL of leaf extract was mixed with DPPH solution thoroughly, vortexed and the total reaction mixture (3mL) was incubated at dark for 30 min. Next, absorbance was read at 517 nm. Ascorbic acid (Merck, India) was used as a standard. This experiment was performed in triplicate.

DPPH free radical scavenging property, expressed in ascorbic acid equivalents and inhibition percentages, was determined by the following formula: ^38, 39^

DPPHinhibition(%)=(ControlOD−SampleOD)/ControlOD*100

***H _2_O _2_ free radical scavenging capacity assay.*** Hydrogen peroxide (H _2_O _2_, SD Fine-Chem, Mumbai, India) free radical scavenging ability was measured according to the method of Ruch *et al.* (1989) in triplicate. The absorbance was taken at 230 nm. Gallic acid was used as standard.

 H _2_O _2_ free radical scavenging capacity was expressed in gallic acid equivalents and inhibition percentages, which were calculated by the formula: ^40, 41^

$${\text{H}}_{\text{2}}{\text{O}}_{\text{2}}\;\text{inhibition}\;\left(\%\right)=\left(\text{Control}\;\text{OD}-\text{Sample}\;\text{OD}\right)/\text{Control}\;\text{OD}\;*100$$

The IC _50_ values of standards and leaves extracts were also calculated for above three free radical scavenging assays.

***β-carotene bleaching test.*** The β-carotene bleaching test antioxidant activity (lipid peroxidation) of the aqueous and 70% ethanolic extracts was done by the method of Minh *et al*. with modifications ^42^. In brief, β-carotene (2 mg) (HiMedia, India) was dissolved in 10 ml of chloroform (Merck, India) and then 1 ml of the chloroform solution was added to 20 μl of linoleic acid (HiMedia-GRM10250, India) and 200 mg of Tween-80 (HiMedia-PCT1513, India). The mixture was evaporated at 50°C, and then 50 ml oxygenated water was added and shaken to form an emulsion. A total of 0.012 ml of leaf extracts were mixed with 0.1 ml of the emulsion. The reactions were left at 50°C, and the absorbance was read at 492 nm. All reactions were read at zero time and every 15 min up to 180 min. This experiment was performed in triplicate. Lipid peroxidation inhibition (LPI) values were expressed using the following formula:

$$\text{LPI}\left(\%\right)=100\times \left[1-\left({\text{A}}_{0}-{\text{A}}_{\text{t}}\right)/\left({\text{A}}_{00}-{\text{A}}_{\text{tt}}\right)\right]$$

Where A _0_ and A _00_ is the absorbance measured at the beginning of the incubation for sample and control, respectively. A _t_ and A _tt_ are the absorbance measured after the incubation of 180 min for sample and control, respectively. Higher LPI value indicates the higher antioxidant activity ^42, 43^.

***Polyphenol oxidase assay.*** The polyphenol oxidase (PPO) assay was carried out in triplicate by using the standard protocol of Esterbauer *et al.* ^44^ A total of 500 mg of fresh leaves was crushed in 2 ml extraction medium containing HCl (Merck, India), sorbitol (Himedia-MB066, India) and NaCl. In the test cuvette, 2.5 ml phosphate buffer was mixed with 0.3 ml of catechol (Loba Chemie, UN281, India) solution. The spectrophotometer was set at 495 nm. Next, 0.2 ml of extract was added, and the change in absorbance was recorded every 30 seconds up to 5 min. PPO activity was expressed as a change in absorbance at 495 nm per minute/g fresh weight ^44, 45^.

***Evaluation of antimicrobial activity.*** Antimicrobial activity was determined using the standard Kirby-Bauer disc diffusion method. A total of 40 μl of the extracts was placed into the paper disc with sterile double distilled water (70% ethanol used as a negative control). After diffusion of the extract, the plates were incubated at 37°C for 16-18 h. Then the zone of inhibition was measured ^14, 15, 46^. Four gram-positive and four gram-negative bacteria (namely, *Bacillus subtilis*, *Bacillus cereus*, *Staphylococcus aureus*, *Staphylococcus epidermidis* and *Escherichia coli*, *Vibrio cholera*, *Pseudomonas aeruginosa*, *Klebsiella pneumonia*, respectively) were used for assessing antimicrobial activity against the negative control double distilled water and 70% ethanol. In the antimicrobial activity study, concentration (µg) of total bioactive compounds in 40 µl was also calculated from the extractive values of the particular solvents.

***Evaluation of *in vitro* anti-diabetic activity (α-amylase inhibition assay).*** The α-amylase activity was measured using a colorimetric method with 3, 5-dinitrosalicylic acid (DNS) reagent. α-amylase (HiMedia, India) inhibition activity was measured using a standard protocol ^16– 18^. The absorbance was measured at 540 nm. Acarbose (Sigma-Aldrich, USA) was used as a positive control. The experimental measurements were performed in triplicate.

The percentage of α-amylase inhibition was determined by using the following formula:

α-amylaseinbition(%)=(ControlOD−SampleOD)/ControlOD×100

The concentration of inhibitors required for inhibiting 50% of the enzyme activity under the assay conditions was presented as the IC _50_ value.

***Evaluation of *in vitro* anti-arthritic activity (inhibition of protein denaturation).*** *In vitro* anti-arthritic activity of extracts was done using a standard protocol, assessing the percentage inhibition of protein denaturation. The experiment was carried out by taking sodium diclofenac (Tokyo Chemical Industry, Japan) as the standard. Absorbance was measured at 416 nm. The experimental measurement was performed in triplicate.

Protein denaturation inhibition percentages were determined by applying the following formula:

Proteindenaturationinhibition(%)=(ControlOD−SampleOD)/ControlOD*100

The IC _50_ values of standard and extracts were also calculated ^19– 21^.

***Evaluation of *in vitro* anti-lithiatic activity (percentage dissolution of calcium oxalates).*** Evaluation of *in vitro* anti-lithiatic activity by percentage dissolution of calcium oxalates was done using the standard method ^22– 24^ in triplicate with slight modifications. The complete procedure is comprises three different steps, namely (i) Experimental kidney stone (calcium oxalate stones) preparation, (ii) Preparation of semi-permeable membrane from chicken eggs, and (iii) Estimation of calcium oxalate by titration method. All the experimental protocols are given below.

**Experimental kidney stone (calcium oxalate stones) preparation**

An equimolar (100 mM) solution of calcium chloride dehydrate (SRL-7065, India), in double-distilled water, and disodium oxalate (Merck, India) in 10 ml of 2 N H _2_SO _4_ (Merck, India) was kept to react with distilled water in a beaker. The resulting precipitate was calcium oxalate. The precipitate was freed from traces of H _2_SO _4_ using 10% ammonia solution and it was washed with double distilled water and dried at 70°C for 5 h.

**Preparation of semi-permeable membrane from chicken eggs**

Shell was removed chemically by placing the chicken eggs, obtained from a local market, in 10% Glacial acetic acid (Merck, India) for 48 h, which caused complete decalcification. The egg membrane washed thoroughly with distilled water, and put into a 10% ammonia solution and stored in a refrigerator at a pH of 7.4.

**Estimation of calcium oxalate by titration method**

The dissolution percentage of calcium oxalate was evaluated by taking precisely 10 mg of calcium oxalate and 10 mg of the lyophilized extracts/standard (1:1 ratio), packed together in the semi-permeable membrane of the egg. This was allowed to suspend in a conical flask containing 100 ml of 0.1 M Tris buffer (SRL-2049170, India). The 1 ^st^ group was set as blank containing only 10 mg of calcium oxalate. The 2 ^nd^ group was established as a positive control containing 10 mg of calcium oxalate along with 10 mg of the standard drug cystone (The Himalaya Drugs Company, Bangalore, India). The 3 ^rd^ and 4 ^th^ groups, along with 10 mg of calcium oxalate, contained aqueous and 70% ethanol extracts, respectively. The conical flasks of all the groups were placed in an incubator and preheated to 37°C for 3 h. The contents of semi-permeable membranes were removed from each group into separate test tubes, and 2 ml of 1 N H _2_SO _4_ was added to each test tube and titrated with 0.9494 N KMnO _4_ (Avantor, Maharashtra, India), till a light pink colour endpoint was obtained. In the calculation 1 ml of 0.9494 N KMnO _4_ is counted as equivalent to 0.1898 mg of 4 Calcium. The amount of remaining undissolved calcium oxalate is subtracted from the total quantity used in the experiment, in the beginning, to know the total amount of calcium oxalate dissolved by various solvent extracts ^22– 24^.

***Evaluation of *in vitro* cytotoxicity.*** A healthy male Wistar rat was purchased from Chittaranjan National Cancer Research Institute, Kolkata, West Bengal and maintained subsequently for the experiments were fed with hind liver pellet or equivalent and water *ad libitum* in 12 h light and dark cycle and at 30°C throughout the experimental period. The experiments were performed under the institutional ethical permission (IAEC/CU/BIOCHEM/SM (1) dated 14.12.2012) and followed the ‘Principles of Laboratory Animal Care’ (NIH publication no. 85-23, revised in 1985). The experiment was performed in triplicate. To conduct the study, a standard rat PBMC isolation technique ^47^ and Cell Viability Assay ^48^ was used. PBMC isolation techniques and Cell Viability Assay are given below in details.

**Leaf extract preparation**

A total of 100 mg dry weights of each plant leaves were mixed, vortexed in 1 ml of double distilled water and 70% ethanol separately, left overnight, centrifuged and supernatant taken.

**Rat PBMC isolation**

A healthy male Wister rat (160 g) was deeply anaesthetized with sodium pentobarbital (50 mg/kg body weight) followed by thoracotomy. Terminal cardiac puncture was made in ventricle using an appropriate sterile needle and blood drawn slowly and collected in PBS-EDTA solution, diluted by 1X PBS in 1:1 ratio and laid on Histopaque-1077 (HiMedia, India), a density gradient cell separation medium of ficoll and sodium diatrizoate was fixed and adjusted with a density of 1.077 g/ml, an equal volume of media and diluted blood. This Histopaque overlaid with diluted blood was centrifuged at 300g at 25°C for 30 mins. The RBCs were a thick red precipitate at the bottom of centrifuge tube, whereas the blood PBMC were found at the interface of Histopaque and the top layer of serum. PBMCs from the interphase were collected, washed twice with PBS, resuspended in 1 ml of DMEM media and this placed in Neubauer Improved Chamber (Marienfeld, Germany) and observed under Microscope Nikon Eclipse TS 100 (Nikon Corporation, Japan) to measure cell concentration.

**Cell viability assay**

A cell viability assay has been done with Trypan Blue. Cells were then laid in 12 well plates at a concentration of 3.6x10 ^7^ cells/ml with a volume of 1 ml at each well except media (DMEM) control containing 1% penicillin-streptomycin (P/S, Antibiotic Antimycotic Solution 100X, Himedia, India) along with 10% FBS and incubated in the CO _2_ incubator for 24 h in 5% CO _2_ humified environment at 37ºC (CO _2_ Incubator Galaxy 48S, New Brunswick, Germany). Next, 10, 5 and 2.5 µl of each of the extracts (different dose of aqueous and ethanolic extracts) were added to the wells except controls and continued in culture for another 48 hrs. Culture plates were then taken out and treated with 10 μl of 0.4% freshly prepared trypan blue solution for 5 mins and readily observed under Nikon Eclipse TS 100 Inverted Phase-contrast Microscope and documented by CCD Camera (DS-Fi2-U3) and NIS-BR Software (Nikon Corporation, Japan).

### Statistical analysis

All the experimental measurements were performed in triplicate (except HPLC-DAD and antimicrobial activity study) and expressed as the average of the three analyses ±standard deviations (SD). The correlation coefficient between variables, means, standard deviations, standard errors, standard curve, IC _50_ values and one-way ANOVA followed by Bonferroni’s post hoc test was calculated by using MS Excel 2007 Software (Microsoft Corporation, Redmond, WA, USA). Figures are prepared in Origin Pro 8 Software (Northampton, MA, USA). A P-value <0.05 was considered as statistically significant.

## Results

### Phytochemical analysis

**Figure 1.**
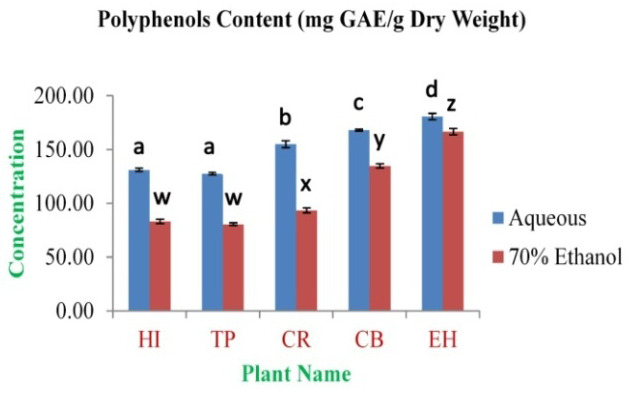
Total Polyphenols Content (mg GAE/g Dry Weight). In the figure different lower case letters (a, b, c, d, e, x, y, z, w, v) in the bars indicates significant differences among means (P<0.05). The blue and red colour bars indicate the aqueous and 70% ethanolic extracts, respectively. X-axis denotes the plant name and Y-axis denotes the concentrations of total polyphenols contents (mg Gallic Acid Equivalent/g Dry Weight).

**Figure 2.**
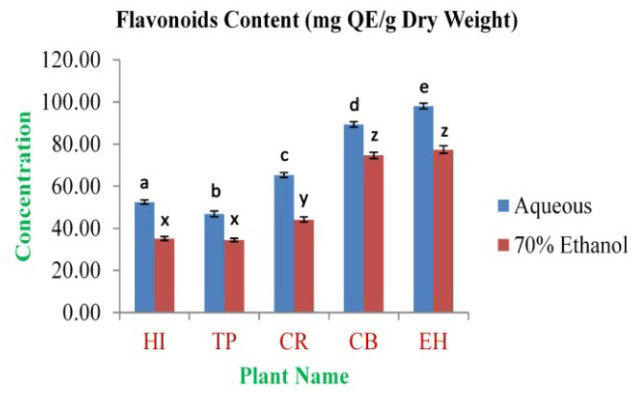
Total flavonoids content (mg QE/g dry weight). In the figure different lower case letters (a, b, c, d, e, x, y, z, w, v) in the bars indicates significant differences among means (P<0.05). The blue and red colour bars indicate the aqueous and 70% ethanolic extracts, respectively. X-axis denotes the plant name and Y-axis denotes the concentrations of total flavonoids contents (mg Quercetin Equivalent/g Dry Weight).

**Figure 3.**
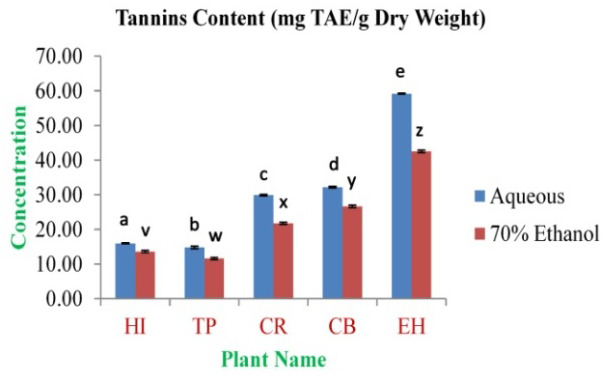
Total tannin content (mg TAE/g dry weight). In the figure different lower case letters (a, b, c, d, e, x, y, z, w, v) in the bars indicates significant differences among means (P<0.05). The blue and red colour bars indicate the aqueous and 70% ethanolic extracts, respectively. X-axis denotes the plant name and Y-axis denotes the concentrations of total tannins contents (mg Tannic Acid Equivalent/g Dry Weight).

The highest concentration of polyphenolics was obtained from EH aqueous and 70% ethanolic extracts (180.59±2.99 and 166.47±3.03 mg GAE/g dry weight, respectively). The lowest concentration was observed in TP aqueous and 70% ethanolic extracts (127.52±1.26 and 80.51±1.21 mg GAE/g dry weight, respectively) ( Figure 1). The highest concentration of flavonoids was obtained from EH aqueous and 70% ethanolic extracts (98.05±1.30 and 77.39±1.79 mg QE/g dry weight, respectively). The lowest concentration was observed in TP aqueous and 70% ethanolic extracts (46.78±1.34 and 34.47±0.81 mg QE/g dry weight, respectively) ( Figure 2). The highest tannin concentration was obtained from EH aqueous and 70% ethanolic extracts (59.21±0.15 and 42.50±0.37 mg TAE/g dry weight, respectively). The lowest concentration was observed in TP aqueous and 70% ethanolic extracts (14.83±0.37 and 11.61±0.32 mg TAE/g dry weight, respectively) ( Figure 3).

**Figure 4.**
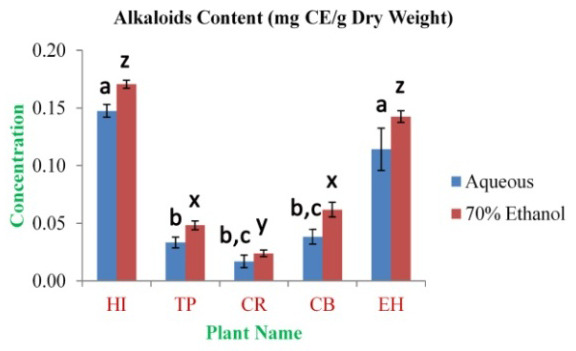
Total alkaloids content (mg CE/g dry weight). In the figure different lower case letters (a, b, c, d, e, x, y, z, w, v) in the bars indicates significant differences among means (P˂0.05). The blue and red colour bars indicate the aqueous and 70% ethanolic extracts, respectively. X-axis denotes the plant name and Y-axis denotes the concentrations of total alkaloids contents (mg Caffeine Equivalent/g Dry Weight).

**Figure 5.**
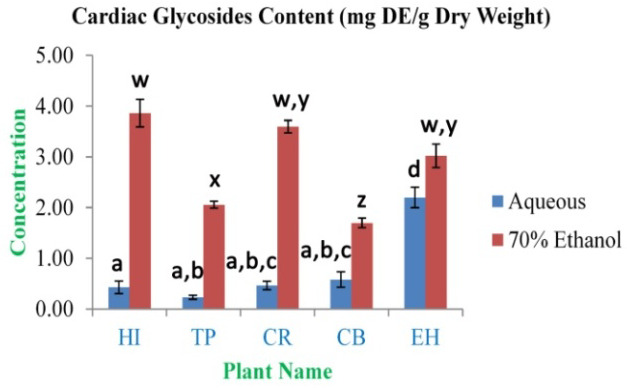
Cardiac glycosides content (mg DE/g dry weight). In the figure different lower case letters (a, b, c, d, e, x, y, z, w, v) in the bars indicates significant differences among means (P˂0.05). The blue and red colour bars indicate the aqueous and 70% ethanolic extracts, respectively. X-axis denotes the plant name and Y-axis denotes the concentrations of total cardiac glycosides contents (mg Digoxin Equivalent/g Dry Weight).

**Figure 6.**
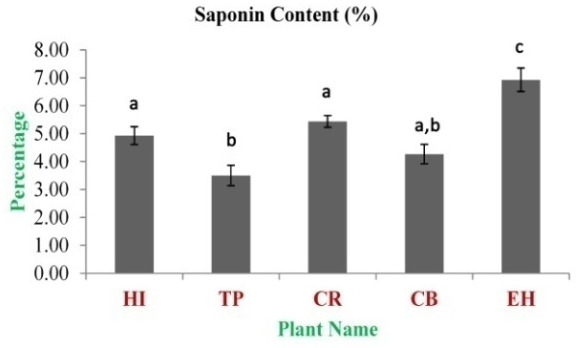
Total saponin content (%). In the figure different lower case letters (a, b, c, d, e, x, y, z, w, v) in the bars indicates significant differences among means (P˂0.05). The gray colour bars indicate the 20% ethanolic extracts. X-axis denotes the plant name and Y-axis denotes the concentrations of total saponin contents (%).

The highest concentration of alkaloid content was observed in HI aqueous and 70% ethanolic extracts (0.148±0.006 and 0.171±0.004 mg CE/g dry weight, respectively). The lowest concentration was observed in CR aqueous and 70% ethanolic extracts (0.017±0.006 and 0.024±0.003 mg CE/g dry weight, respectively) ( Figure 4). The highest concentration of cardiac glycosides content was observed in EH aqueous and HI 70% ethanolic extracts (2.20±0.20 and 3.86±0.27 mg DE/g dry weight, respectively). The lowest concentration was obtained from TP aqueous and CB 70% ethanolic extracts (0.23±0.04 and 1.70±0.09 mg DE/g dry weight, respectively) ( Figure 5). The total saponin content for these medicinal herbs dried powdered leaves 20% ethanolic extracts was estimated. The highest concentration was observed in EH (6.93±0.42%). The lowest concentration was observed in TP (3.50±0.36%) ( Figure 6).

### Identified compounds from the HPLC-DAD profile

**Figure 7.**
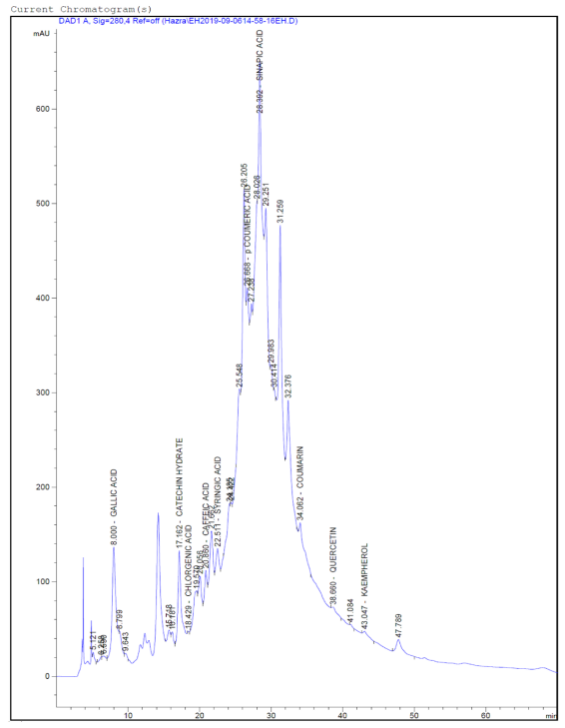
HPLC-DAD chromatogram of the phenolic compounds presents in the EH 70% ethanolic extract (representative figure). Polyphenolic compounds were separated by Phenomenex-C18 (2)-column, the Diode Array Detector was set at 280 nm; the mobile phase consisted of 3% acetic acid water and acetonitrile. The flow rate was 0.9 ml/min in gradient conditions. In the figure X-axis denotes the units of time (minutes) i.e. retention time (tR) and Y axis denotes the intensity of absorbance (milli-Absorbance units) or concentration. Ten compounds were detected in chromatogram.

The analysis of HPLC-DAD profile obtained from the plants leaves 70% ethanolic extracts identified ten bioactive phenolic compounds GA, CH, CHA, CA, SYA, pCA, SIA, CM, QE and KMP ( Figure 7 and *Extended data*, Figures S1, S2, S3, S4) ^49^. Among these three compounds were present in all the extracts (chlorogenic acid, coumaric acid and coumarin). In EH and TP highest ten compounds were identified. In HI lowest six compounds were identified. In EH six compounds were found in the highest amount, and these are GA (83.258 ng/µl), CH (122.149 ng/µl), SYA (9.487 ng/µl, pCA (10.017 ng/µl, SIA (297.315 ng/µl, CM (3.719 ng/µl). In each of TP and CR two phenolic acids were found in the highest amount and these are CHA (6.405 ng/µl), KMP (5.241 ng/µl) and CA (23.006 ng/µl), QE (15.099 ng/µl), respectively. Sinapic acid was the most abundant phenolic acid present in all the leaves extracts as well as it is found in the highest amount in EH extract. Availability of coumarin among these standard phenolics is found minimum in all the extracts. The HPLC-DAD profile showed in chromatogram representation and in Table 1.

**Table 1.** HPLC-DAD-based quantification of different bioactive compounds present in the extracts.

Compound Name	Plant Name and Amount [ng/µl]				
	HI	TP	CR	CB	EH
Gallic acid	ND	9.991	13.557	8.682	83.258
Catechin hydrate	1.382	1.392	ND	ND	122.149
Chlorogenic acid	4.552	6.405	1.334	2.192	1.332
Caffeic acid	ND	0.882	23.006	0.991	10.104
Syringic acid	2.356	1.601	ND	4.627	9.487
Coumeric acid	0.891	0.383	3.515	0.087	10.017
Sinapic acid	1.197	2.714	21.899	14.613	297.315
Coumarin	0.009	1.254	1.221	0.000	3.719
Quercetin	ND	1.929	15.099	1.682	3.303
Kaempherol	ND	5.241	ND	3.281	4.330

### *In vitro* antioxidant activity

**Figure 8.**
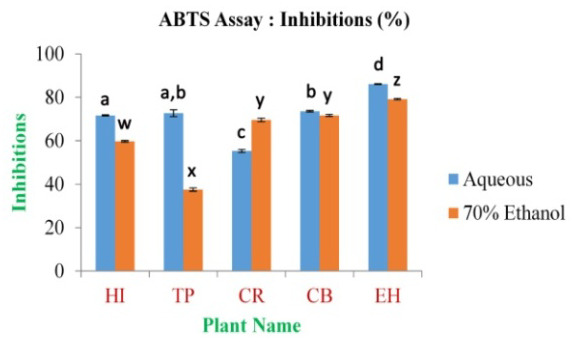
Inhibition percentage of extracts by ABTS free radical scavenging assay. In the figure different lower case letters (a, b, c, d, e, x, y, z, w, v) in the bars indicates significant differences among means (P˂0.05). The blue and red colour bars indicate the aqueous and 70% ethanolic extracts, respectively. X-axis denotes the plant name and Y-axis denotes the inhibition percentages by the extracts (%).

In the present study, the maximum inhibition percentage of ABTS free radical scavenging assay was found to be 86.10±0.22% and 79.19±0.36% for EH aqueous and 70% ethanolic extract, respectively. The minimum is 55.33±0.64% and 37.57±0.80% for CR and TP in aqueous and 70% ethanolic extract ( Figure 8 and *Extended data*, Figure S5) ^49^, respectively; the inhibition percentage for standard ascorbic acid was found to be 98.29% at 0.5 mg/ml of concentration.

**Figure 9.**
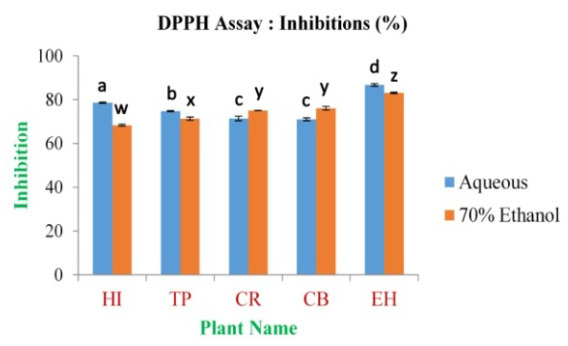
Inhibition percentage of extracts by DPPH free radical scavenging assay. In the figure different lower case letters (a, b, c, d, e, x, y, z, w, v) in the bars indicates significant differences among means (P˂0.05). The blue and red colour bars indicate the aqueous and 70% ethanolic extracts, respectively. X-axis denotes the plant name and Y-axis denotes the inhibition percentages by the extracts (%).

The study reveals that the maximum inhibition percentage of DPPH free radical scavenging assay was 86.75±0.56% and 83.07±0.25% for the EH aqueous and 70% ethanolic extract, respectively. The minimum was 71.02±0.07% and 68.34±0.41% for CB and HI in aqueous and 70% ethanolic extract ( Figure 9 and *Extended data*, Figure S6) ^49^, respectively; this compared with the percentage inhibition of ascorbic acid which was 92.77% at 0.5 mg/ml of concentration in the present study.

**Figure 10.**
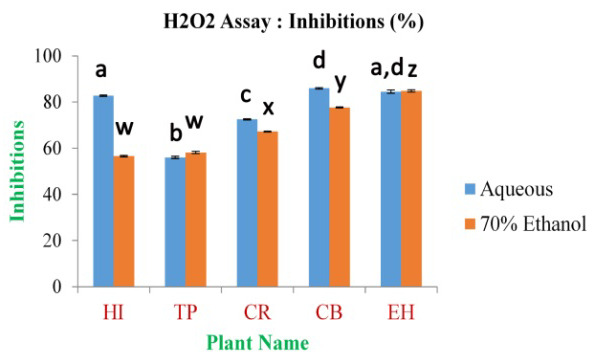
Inhibition percentage of extracts by H _2_O _2_ free radical scavenging assay. In the figure different lower case letters (a, b, c, d, e, x, y, z, w, v) in the bars indicates significant differences among means (P˂0.05). The blue and red colour bars indicate the aqueous and 70% ethanolic extracts, respectively. X-axis denotes the plant name and Y-axis denotes the inhibition percentages by the extracts (%).

The maximum inhibition percentage of H _2_O _2_ free radical scavenging assay was found to be 85.92±0.31% and 84.83±0.46% for CB aqueous and EH 70% ethanolic extract, respectively. The minimum is 56.05±0.48% and 56.57±0.36% for TP and HI in aqueous and 70% ethanolic extract ( Figure 10 and *Extended data*, Figure S7) ^49^, respectively; the inhibition percentage for standard Gallic acid was 95.10% at 0.5 mg/ml of concentration.

**Figure 11.**
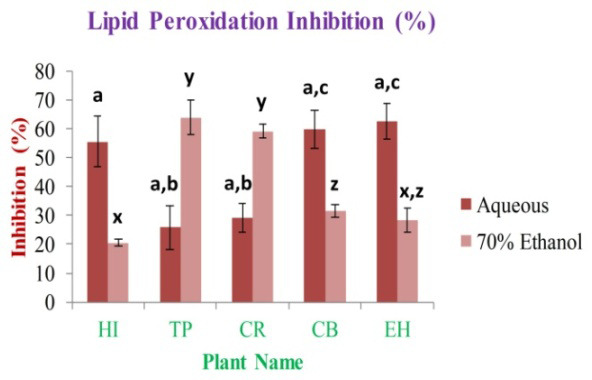
Lipid peroxidation inhibition activity of extracts (%). In the figure different lower case letters (a, b, c, d, e, x, y, z, w, v) in the bars indicates significant differences among means (P˂0.05). The red and pink colour bars indicate the aqueous and 70% ethanolic extracts, respectively. X-axis denotes the plant name and Y-axis denotes the inhibition percentages by the extracts (%).

In this investigation, the antioxidant activity of lipid peroxidation (LPI) by ß-carotene bleaching test concluded that the highest activity was observed in case of EH aqueous and TP 70% ethanolic extracts, and it is 62.58±6.00% and 63.89±5.87%, respectively. The lowest activity was observed in the case of TP aqueous and HI 70% ethanolic extracts, and it is 25.85±7.51% and 20.44±1.24%, respectively ( Figure 11).

### PPO

**Figure 12.**
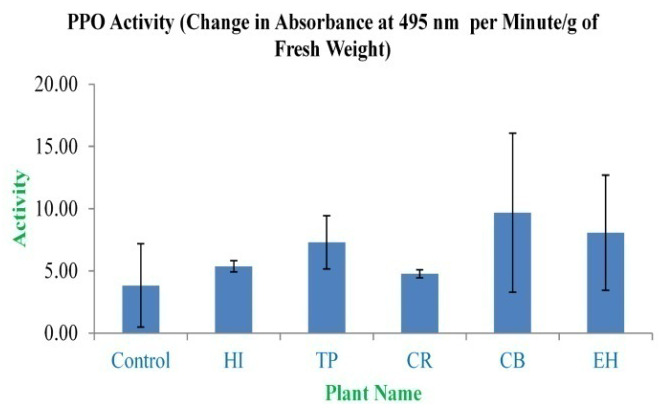
Polyphenol oxidase activity assay. In the figure the bars indicates no significant differences among means (P˃0.05). X-axis denotes the plant name and Y-axis denotes the change in absorbance at 495 nm per minute/g of fresh weight by the extracts (%).

In the study, the enzyme PPO activity assay concluded that the highest and lowest activity was observed in CB extracts (9.67±6.39) and CR extracts (4.77±0.32), respectively, in terms of change in absorbance at 495 nm per minute/g of fresh weight, which is greater than the control used in the PPO activity assay, i.e. 3.83±3.35 ( Figure 12).

### *In vitro* antimicrobial activity

Zone of inhibition shown by aqueous and 70% ethanolic leaves extract indicated the presence of high antimicrobial activity. EH aqueous extracts showed the highest zone of inhibition in comparing with all the extracts. TP and CR 70% ethanolic extracts had given better antimicrobial properties than other leaves 70% ethanolic extracts. SE strains have not given any zone of inhibition in case of all the extracts ( Table 2 and *Extended data*, Figures S8, S9, S10, S11) ^49^. The concentration (µg) of bioactive compounds in 40 µl which was the given dose for the assay in antimicrobial activity study was determined from the extractive values of the aqueous and 70% ethanolic leaf extracts ( *Extended data*, Table S1) ^49^.

**Table 2.** Zone of inhibitions of leaves extracts against different bacterial strains.

Strains	Zone of Inhibition (mm)													
	BC		BS		EC		KP		PA		SA		VC	
Solvents →	A	E	A	E	A	E	A	E	A	E	A	E	A	E
Plant Name ↓														
HI	9.33	Nil	10.67	6.67	14.67	0	14.67	Nil	11.33	4.67	16.67	7	14.67	11.33
TP	11.33	10.67	9.67	5	13.33	2.67	9.67	-1.67	11.33	5.67	16.67	3.67	14	9.33
CR	5	7	10.67	0.67	10.67	5	11.33	-1	10.33	6.67	10.67	7	13.33	13.67
CB	10.67	7.33	10.67	-2.67	17	5	15.33	-0.67	18.33	7.67	14.67	7	20	11.33
EH	15.33	7	14.33	-0.33	20.33	5	18.67	-0.33	20.33	2	19.67	7	19	11.67

### *In vitro* anti-diabetic activity

**Figure 13.**
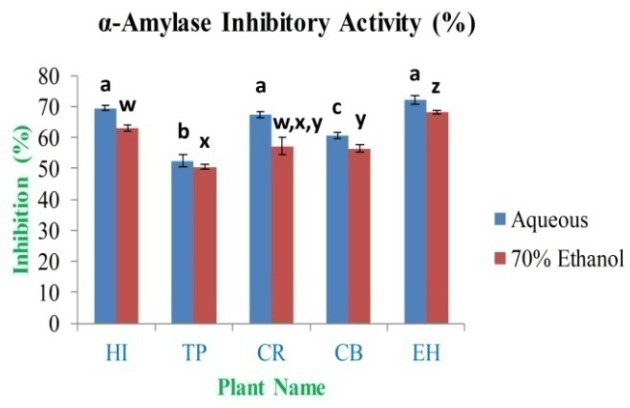
α-amylase inhibitory activity (%) of extracts. In the figure different lower case letters (a, b, c, d, e, x, y, z, w, v) in the bars indicates significant differences among means (P˂0.05). The blue and red colour bars indicate the aqueous and 70% ethanolic extracts, respectively. X-axis denotes the plant name and Y-axis denotes the α-amylase inhibition percentages by the extracts (%).

In the current study, different concentrations of extract were selected for the *in vitro* assay between 10 mg/ml and 0.0195 mg/ml. Based on the results, the maximum α-amylase inhibition percentage was found to be 72.19±1.26% and 68.14±0.71% for EH aqueous and 70% ethanolic extract, respectively. The minimum inhibition activity is 52.54±1.94% and 50.48±0.82% for TP aqueous and 70% ethanolic extract ( Figure 13), respectively; this compared to an inhibition percentage for standard acarbose of 99.00% at 10 mg/ml of concentration.

### *In vitro* anti-arthritic activity

**Figure 14.**
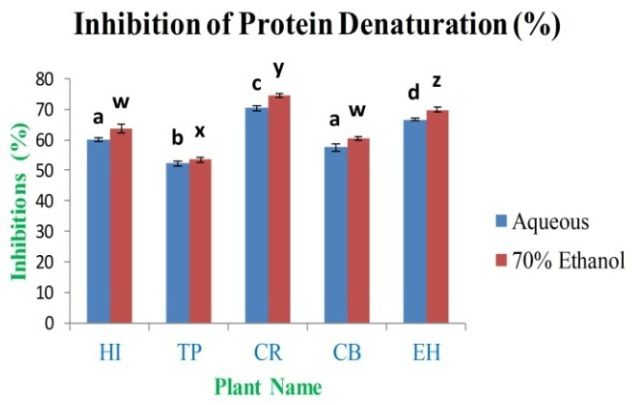
Percentage (%) inhibition of protein denaturation by extracts. In the figure different lower case letters (a, b, c, d, e, x, y, z, w, v) in the bars indicates significant differences among means (P˂0.05). The blue and red colour bars indicate the aqueous and 70% ethanolic extracts, respectively. X-axis denotes the plant name and Y-axis denotes the inhibition percentages of protein denaturation by the extracts (%).

The anti-arthritic activity of aqueous and 70% ethanolic extracts was determined by the *in vitro* models, i.e. inhibition of protein denaturation. The various concentrations of extracts were selected for the assay between 1 mg/ml and 0.00196 mg/ml. The study observed that the maximum inhibition percentage was 70.36±0.69% and 74.54±0.75% for CR aqueous and 70% ethanolic extracts, respectively. The minimum inhibition activity is 52.28±0.91% and 53.48±0.89% for TP aqueous and 70% ethanol extract ( Figure 14), respectively, as compared to inhibition percentage for standard diclofenac sodium was 97.25% at 1 mg/ml of concentration.

### *In vitro* anti-lithiatic activity

**Figure 15.**
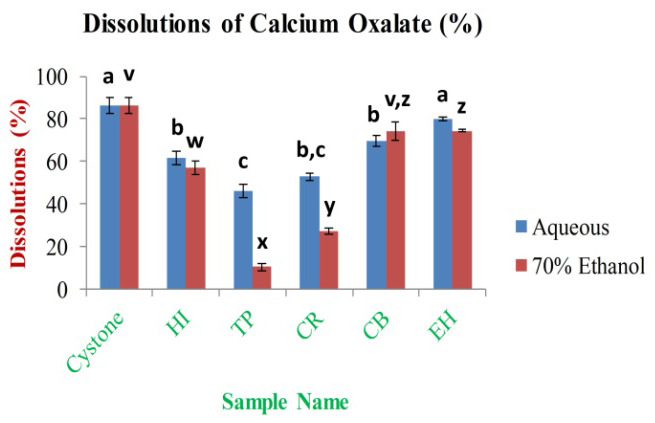
Percentage (%) dissolution of calcium oxalate by extracts. In the figure different lower case letters (a, b, c, d, e, x, y, z, w, v) in the bars indicates significant differences among means (P˂0.05). The blue and red colour bars indicate the aqueous and 70% ethanolic extracts, respectively. X-axis denotes the sample name and Y-axis denotes the percentage (%) dissolution of calcium oxalate by extracts.

In the research investigation, titration was used to assess the *in vitro* anti-lithiatic property of aqueous and 70% ethanolic extracts using a calcium oxalate dissolution assay. Based on the results obtained in the study, the highest dissolution percentage of calcium oxalate was 79.98±0.95% and 74.41±4.43% for EH aqueous and CB 70% ethanolic extract, respectively. The lowest is 46.37±3.22% and 10.45±1.78% for TP aqueous and 70% ethanolic extract ( Figure 15), respectively. The dissolution percentage of calcium oxalate for the standard drug cystone was 86.42±3.57% at 1 mg/ml of concentration.

**Figure 16.**
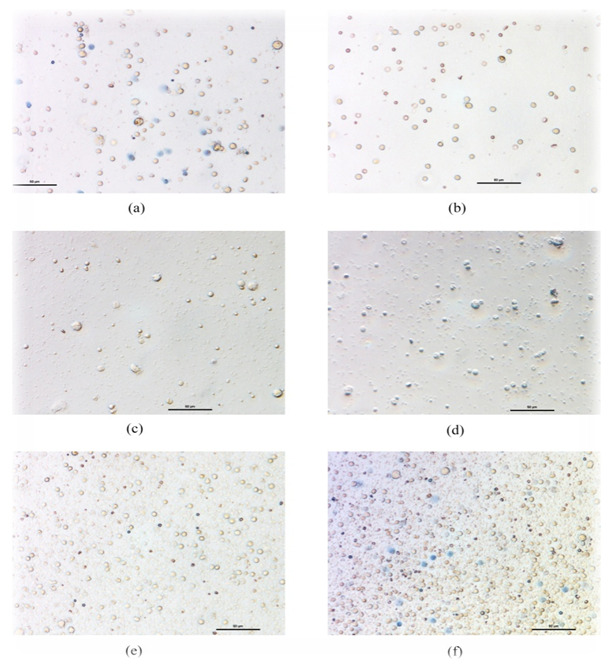
Representative figures showed the cells with Trypan Blue (microphotographs). **a**. Cell control after 48 hrs of seeding; **b**. Cells after seeding with drugs; **c**. EH aqueous drug effect after 48 hrs; **d**. EH 70% ethanolic drug effect after 48 hrs; **e**. CR aqueous drug effect after 48 hrs; **f**. CR 70% ethanolic drug effect after 48 hrs; The given dose of extracts is 10 μl.

### *In vitro* cytotoxicity study

In the trypan blue exclusion assay, the absolute count of total cells in each field vs cells with characteristic trypan blue stains were counted for each plant extract-treated group. In our study, 2.5 µl and 5 µl dose of crude drugs did not show any cytotoxic effects towards PBMC. However, 10 µl of the dose showed the effects ( Figure 16a–f).

**Figure 17.**
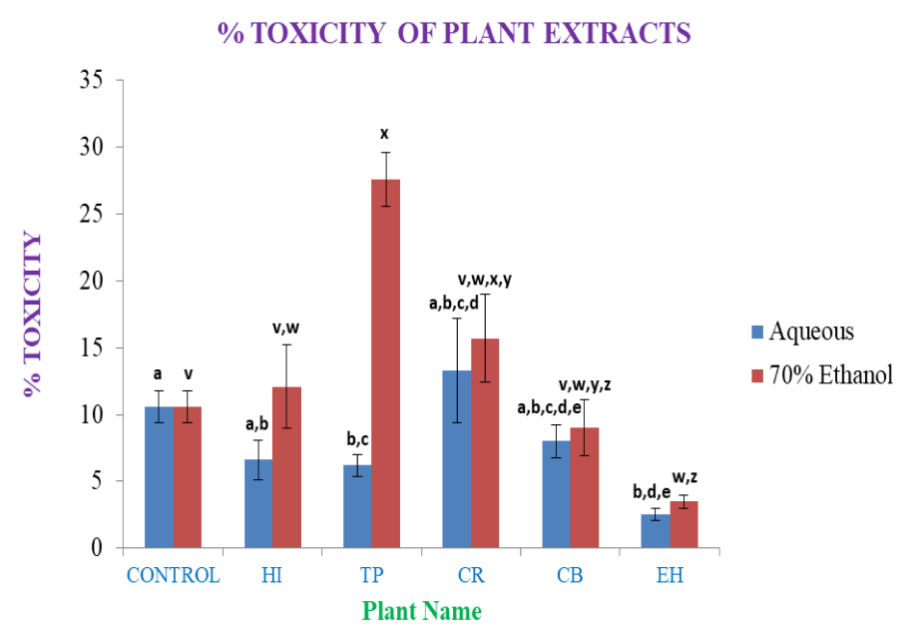
Cytotoxic effect of plant leaves extracts (%). In the figure different lower case letters (a, b, c, d, e, x, y, z, w, v) in the bars indicates significant differences among means (P˂0.05). The blue and red colour bars indicate the aqueous and 70% ethanolic extracts, respectively. X-axis denotes the sample name and Y-axis denotes percentage (%) of toxicity of the plants leaves extracts towards PBMC at the dose of 10μl.

The dose-dependent (10 µl of crude plant extract) cell viability assay elicited a 10-12% mortality rate of PBMC in 48 hrs of culture in the control; only the aqueous extracts of CR showed higher mortality than control, with other aqueous extracts eliciting lower mortality rates, showing some nontoxic or cytoprotective properties for PBMC or immune cells ( Figure 17). However, the EH aqueous extract showed maximum cytoprotectivity showing a mortality of ~2.5%. EH 70% ethanolic extracts showed cytoprotectivity (~4%) nearly comparable to its aqueous soluble counterpart. The 70% ethanolic extracts showed higher mortality in PBMC. Particularly, CR and TP 70% ethanolic extracts are much cytotoxic, where TP found over two-fold cytotoxicity than control. Both extracts of CB showed cytoprotectivity compared to the control.

From the above results, it is observed that except CR aqueous extracts the aqueous medium is overall cytoprotective. Most of the drugs in this form are less cytotoxic than control; hence it is much preferred as a medium in pharmaceutical application purposes. EH aqueous and 70% ethanolic extracts has been found much more cytoprotective to PBMCs. Hence it can be used to treat against immune disorders after necessary processing.

Raw data of each experiment performed are available as *Underlying data* ^50^.

## Discussion

The oxidation reactions and free radical production lead to some oxidative stress-related disorders such as diabetes, arthritis, urolithiasis and cancer. The activity of phenolic compounds against oxidative stress-related mechanism can have substantial therapeutic applications in the pharmaceutical and nutraceutical industry ^12, 34, 51– 54^.

Flavonoids are the category of plant secondary metabolites with high antioxidant, antimicrobial and chelating activities. Antioxidant activities of flavonoids depend on the structure and substitution pathways of hydroxyl groups. The antioxidative properties of flavonoids classes are due to various reaction mechanisms, such as scavenging of free radicals, chelation of metal ions (e.g. iron and copper), and inhibition of enzymes which are responsible for free radical production. Depending on their specific structure, it can inhibit or regulate all the possible ROS, which can cause cellular damages. Previous studies have concluded the protective ability of flavonoids against various diseases ^53, 55, 56^.

Tannins are found in the stem and barks of various plants rather than in the leaves. The tannins showed excellent antioxidant, antimicrobial and anti-cancer activities. These are astringent, bitter plant polymeric compound which can be capable of tanning leathers, and can sediment proteins, amino acids, alkaloids and nitrogenous substances ^57^. The tannin-protein complex can provide persistent antioxidant and antimicrobial properties ^53, 56, 57^.

Alkaloids are essential secondary plant metabolites, and have extensive applications in present-day herbal formulations as these compounds have huge potentiality as an antimicrobial agent ^29, 56^.

The amount of cardiac glycoside found in extracts is minor compared to larger quantities that have been reported in various plant leaves. This signifies that the leaves are not toxic as glycosides act as sodium-potassium ATPase inhibitor leading to cell death. Cardiac glycosides are used for the treatment of different oxidative stress-related cardiac diseases such as congestive heart failure and cardiac arrhythmia ^11, 30^.

Saponin is an amphipathic (hydrophilic) glycoside moiety with lipophilic triterpene or steroid derivatives ^58^. This is an important bioactive compound which plays a vital role to prevent antimicrobial activity and other biological disorders ^31, 32^. In the study, polyphenolic compounds, flavoinoids, tannins, alkaloids and cardiac glycosides contents showed very significant (p-value <0.01) presence in both the extracts as well as they are positively and variably correlated (Tables S2 and S3) with each other and antioxidant properties, too.

In the current investigation, a simple, accurate, and reproducible online HPLC-DAD method has been used and validated for the identification and quantification of phenolics. Phenolics, especially phenolic acids and flavonoids, are conventional natural products found in plants that have significant antioxidant and anti-inflammatory activity. ROS are produced by various metabolic activities and is a significant contributor to many chronic and oxidative stress-related diseases. Phenolic acids have been demonstrated as potent scavengers of ROS. These are sub-classified as benzoic acid and cinnamic acid backbone structure containing seven (C6-C1) and nine (C6-C3) carbon atoms, respectively. Hydroxybenzoic acid derivatives such as gallic acid, syringic acid and cinnamic acid derivatives like caffeic acid, chlorogenic acid, sinapic acid, p-coumaric acid are widely available in plants ^33, 34, 59, 60^. Masek *et al*., concluded that caffeic acid is the predominantly and widely available phenolic acid, and it exhibits antioxidant and iron-chelating abilities which are better than p coumaric acid ^61^. Cinnamic acid derivative sinapic acid exhibits potent antioxidant, antimicrobial and anti-arthritic activities ^62^. Katada *et al*. showed that hydroxycinnamic acid derivative chlorogenic acid act as a dietary supplement which is highly effective in oxidative stress-related diseases such as arthritis and high blood pressure ^63^.

ABTS free radical scavenging assay serve as assessing the antioxidant property. The IC _50_ analysis is used for estimating the activity of an inhibitor. IC _50_ analysis showed that the standard ascorbic acid is required 0.237 mg/ml, whereas leaf extracts required a higher amount to reach at IC _50_ ( *Extended data*, Table S4) ^49^. ABTS assay is a free radical cation decolourization assay. In the study, potassium persulfate was used to give a stable form of ABTS radical cation ^35– 37^.

In the DPPH assay, IC _50_ analysis showed that the standard ascorbic acid is required 0.257 mg/ml, whereas leaves extracts required a higher amount to reach at IC _50_ ( *Extended data*, Table S5) ^49^. DPPH is a highly used free radical to determine the free radical scavenging activity of natural antioxidants. This free radical scavenging assay is based on the reduction of DPPH radicals in methanol in the presence of hydrogen-donating antioxidant due to the formation of the non-radical shape of DPPH-H. DPPH is unaffected by side reactions, like metal ion chelating and enzyme inhibitions and it is counted as one of the benefits of it. In this method, a change in colour from purple to yellow happens, and this is proportional to the concentration and radical scavenging capacity of the extracts in the form of hydrogen donating capability ^4, 39, 46, 53, 54^.

In the H _2_O _2_ radical scavenging assay, IC _50_ analysis showed that the standard gallic acid is required 0.247 mg/ml, whereas extracts required a higher amount for IC _50_ ( *Extended data*, Table S6) ^49^. H _2_O _2_ becomes toxic to the cell because it is rapidly decomposed into oxygen and water and gives rise to the hydroxyl radical, which can initiate lipid peroxidation mechanisms by obtaining hydrogen atoms from unsaturated fatty acids and cause DNA strands damages. The hydroxyl radical is short-lived severely reactive free radicals, formed in the biological systems, capable of damaging a wide range of biomolecules located in the living cells through diffusion-limited reaction. Its removal is necessary for the protection of food, too ^4, 11, 12, 40, 41^.

It has been well known that carotenoids class of compounds undergo bleaching, i.e. lose their colour, when exposed to free radicals or to oxidative species. This entire mechanism occurs by the addition of the conjugated double bond which is present in the available reaction system either by the help of cleavage or by adding one of the double bonds of molecules. This cleavage occurrence can be identified by analyzing the products that are created, which are primarily carbonyls or epoxides ^43, 44^. Membrane lipids are rich in unsaturated fatty acids which are most susceptible to oxidative mechanisms. The free radical chain reaction is widely accepted as a common process of lipid peroxidation, and it is thought that the inhibition of lipid peroxidation is caused by antioxidants due to their free radical scavenging properties ^13, 55^. Flavonoids, aglycones and glycosides showed good lipid peroxidation activities by the ß-carotene bleaching test ^55^. Phytomolecules such as saponin, flavonoids and tannins found to protect against lipid peroxidation ^64^.

The study showed the presence of significant level of antioxidants in the plant extracts (aqueous and 70% ethanol). The study also showed the variable degrees of correlation ( *Extended data*, Table S3) ^49^ between the phytochemicals content and antioxidant activity. The results of the different radical scavenging assays are found to be positively correlated ( *Extended data*, Table S7) ^49^ with each other, signifying the interplay between different antioxidants and their mode of action in combating cellular stress.

From previous studies and reports, it was observed that there is a positive correlation exists between antioxidant property and the bioactive compounds. It can be concluded that the bioactive compounds appear to be responsible for the significant (p-value <0.01) antioxidant property of both the solvents extracts ^12, 13^. The observation of the investigations agrees with the point that the ethnomedicinal plants are the principal source of therapeutically and nutritionally used natural antioxidants ^52^. The study findings conclude that the aqueous extracts have maximum antioxidant activity in comparison with the 70% ethanolic extracts. EH has huge and intense potentiality towards pharmaceutical and nutraceutical importance due to the presence of high polyphenolic compounds which is comparable with the green and black teas ^65^. Using the above *in vitro* test, the results suggested that EH exhibited potent antioxidant activity, and it can serve as a new source of the natural antioxidant agent ^12, 66^.

PPO is a widely distributed copper-containing enzyme in plants. It is known to be responsible for the enzymatic browning reaction occurring during the handling, storage and processing of the fruits and vegetables. The two molecular oxygen occurs in the two separate reactions when PPO shows the activity. The PPO enzyme catalyzes the oxidation of phenolic compounds into highly reactive quinones. Polymerization of PPO-derived quinones causes the postharvest browning of cut or bruised fruit, but the native physiological functions of PPOs in undamaged, intact plant cells are not well understood ^44, 45, 67, 68^. The p-value <0.05, which showed no significant level of enzyme PPO activity in the experimental extracts. In the present study, variable degrees of correlation ( *Extended data*, Table S8, S9 and S10) ^49^ are found between phytochemicals and free radical scavenging assay with the PPO activity.

The complete results obtained from the antimicrobial activity study indicated that aqueous extracts had high antimicrobial property than the 70% ethanolic extracts, which supports the previous study of Abubakar, 2009 ^69^. The presence of bioactive metabolites in the extracts such as flavonoids, tannins, saponins, glycosides, alkaloids, phenolics, steroids and anthraquinone as reported earlier is likely to be responsible for the good antimicrobial property. Polyphenols and other bioactive molecules regulate the synthesis of nucleic acids of both Gram-negative and Gram-positive bacteria; that is why the proper zone of inhibition has been observed in the assay ^13, 56, 70^. Perumal showed that plants with high caffeic acid levels had shown significant antimicrobial activity. In the study, 70% ethanolic extracts of CR and TP contains the highest amount of caffeic acid, and these two leaves extracts showed better antimicrobial activities which support the previous study as well ^71^. The antimicrobial activity also depends on the ROS formation. Balanced state of the body interrupted by pathological situations due to overproduction of ROS and comparatively low amount of endogenous antioxidants in the body. It results in the reaction between ROS and intra and extracellular species leading to the emergence of oxidative stress-related disorders and pathogenesis. To control oxidative stresses, it is necessary to maintain the balance between ROS and antioxidants by administering exogenous natural antioxidants such as hydroxycinnamic acids ^46, 62^.

The *in vitro* anti-diabetic property evaluation of the plants leaves extracts was assessed by inhibition of polysaccharides degrading enzyme α-amylase inhibitory method. A large variety of α-amylase inhibitors have been reported from various plants, which protect against oxidative damage resulted in hyperglycemia. Inhibition of enzyme activity may be due to the presence of potentially bioactive compounds like polyphenols, alkaloids, flavonoids, tannins and glycosides. The DNS reagent assay is a widely used procedure to quantify the amount of reducing sugars produced after-treatment of the solution by α-amylase with different types of samples ^16– 18^. IC _50_ analysis showed that the standard acarbose is required 3.55 mg/ml, whereas leaves extracts required a higher amount to reach at IC _50_ ( *Extended data*, Table S11) ^49^. Bioactive constituents are the natural source of antioxidants and responsible for preventing the destruction of β-cells and diabetes-induced ROS formation. The anti-diabetic and antioxidant properties of the EH leaves might be due to the synergistic effect of biologically active phytochemicals present in the extracts ^64^.

The *in vitro* anti-arthritic activity investigation by protein denaturation method indicated that all the 70% ethanolic extract had showed maximum inhibition when compared to the aqueous extracts and IC _50_ analysis showed that standard sodium diclofenac is required at 0.430 mg/ml, whereas leaf extracts had higher IC _50_ values ( *Extended data*, Table S12) ^49^. The significant *in vitro* anti-arthritic activity exhibited by the extracts is caused may be due to the presence of flavonoids, polyphenols and tannins. Denaturation of the protein involves the breakdown of chemical structures of the molecules and finally leads to cell death; it happens due to various stresses like a high level of salt, temperature and acidity. The processes of protein denaturation may be related with the alteration in electrostatic, hydrogen, hydrophobic and disulphide bonding. Denaturation of proteins is leading to oxidative stress-related diseases such as inflammatory situations, rheumatoid arthritis, high blood pressure, diabetes and cancer. The results concluded that leaves extracts of CR have the highest ability to control the oxidative stress-related generation of auto-antigen, which relates to the inhibition of the denaturation of proteins ^19– 21^.

The dissolution of calcium oxalate and the number of calcium ions in the solutions are inversely related. The current *in vitro* anti-lithiatic investigation concluded that the aqueous extracts showed higher dissolution percentage of calcium oxalate crystals (except CB) than 70% ethanolic extracts. The result of CB aqueous extract contradicts with the previous reports of Celestian *et al*., who showed that CB aqueous extracts had higher dissolution percentages of calcium oxalates ^23^. According to our knowledge, the present work highlighted the *in vitro* anti-urolithiasis activity of HI, and CR leaves extracts for the first time. Lithiasis and its co-morbidities proceed to the development of oxidative stresses which is known to be a primary reason for inflammation. Previous research suggests that oxidative stresses and inflammation generated by one disorder, and it may happen in a particular situation which produces the development of the co-morbidities. As observed by the researchers that mildly high calcium or phosphate can promote the deposition of calcium phosphate crystals in the renal interstitium with localized swellings and deposition of collagen resulting to the production of Randall’s plaque or it may be possible that mildly high calcium and phosphate or oxalate and low citrate or magnesium levels in the urine can cause to crystallization in the collecting ducts of kidney that is oxidatively stressed and injured ^72^. The present study reveals that the aqueous and 70% ethanolic extracts of EH can be able to dissolve the calcium oxalate stones which are higher comparing with the other leaf extracts. The result of the study concluded the necessity of EH aqueous extracts for the treatment of renal stones which agrees with the previous investigation as well ^73^, whereas the TP extracts contradict with the previous observations ^74^. The p-value˂0.01, which showed a very significant *in vitro* bioactivity in case of extracts using each solvent. In this study, the phytochemicals and antioxidant properties are variably and remarkably correlated with oxidative stress-related medicinal properties ( *Extended data*, Tables S13 and S14) ^49^.

## Conclusions

It is concluded that the medicinal plants are best sources of phytochemicals and remedial agents for several disorders. In the present investigation, the extracts of HI, TP, CR, CB and EH were found to be rich in secondary plant metabolites, which showed significant *in vitro* antioxidant and medicinal properties as well as minimal level of cytotoxicity ^75^. Though, for all the *in vitro* experiments, further studies are recommended to know and evaluate the pharmacological efficacy, potentiality and mode of action. The reasonable difference between the presence of phytochemical contents are may be due to leaves maturity, fertility, pest exposure, moisture, relative water content, pH, solubility, solvents polarity, environmental factors like pollution, solar reflectance, rainfalls, precipitation, location and temperature ^4, 37^.

In conclusions, these results suggested that among these five medicinal weeds, EH has showed the highest bioactive component presence as well as the antioxidant properties which agree with the previous studies, too ^12, 52^. EH showed better *in vitro* medicinal properties and prominent cytoprotectivity on dose dependant study in comparison with the other four weeds due to the presence of high amount of vital phytomolecules such as sinapic acid, gallic acid, caffeic acid or chlorogenic acid ^61– 63^. In future, *Euphorbia hirta* Linn. can be used as an important medicinal plant to isolate and identify the active phytocompounds for the therapeutic and natural antioxidant preparation purposes cost effectively.

Thus, the study has shown the path that traditionally used easily available weeds can be a low-cost source of important bioactive molecules with potential for herbal drug development.

## Data availability

### Underlying data

Figshare: Phytochemical Composition Analysis and Evaluation of In Vitro Medicinal Properties and Cytotoxicity of Five Wild Weeds. https://doi.org/10.6084/m9.figshare.12115827.v2 ^50^.

File ‘F1000 Raw Data Final’ contains the raw output data for each repeat of each experiment.

### Extended data

Figshare: f1000 Extended data (Phytochemistry and Phytopharmacology of 5 Medicinal Weeds). https://doi.org/10.6084/m9.figshare.12205631 ^49^.

File ‘F1000 Extended data’ contains the following extended data:

- Table S1. Concentration (µg) of bioactive compounds in 40µl in antimicrobial activity study.
- Table S2. Correlation between bioactive components.
- Table S3. Correlation between antioxidant assays and bioactive components.
- Table S4. IC _50_ of standard reagent and leaves extracts in ABTS free radical scavenging assay.
- Table S5. IC _50_ of standard reagent and leaves extracts in DPPH free radical scavenging assay.
- Table S6. IC _50_ of standard reagent and leaves extracts in H _2_O _2_ free radical scavenging assay.
- Table S7. Correlation between antioxidant assays.
- Table S8. Correlation between PPO and bioactive compounds.
- Table S9. Correlation between PPO and Saponin.
- Table S10. Correlation between antioxidant assays and PPO.
- Table S11. IC _50_ of standard drug and extracts in α-amylase inhibitory activity.
- Table S12. IC _50_ of standard drug and leaves extracts in inhibition of protein denaturation.
- Table S13. Correlation between bioactive compounds and *in vitro* medicinal properties.
- Table S14. Correlation between antioxidant assays and *in vitro* medicinal properties.
- Figure S1. HI HPLC-DAD chromatogram of the phenolic compounds presents in the 70% ethanolic extract.
- Figure S2. TP HPLC-DAD chromatogram of the phenolic compounds presents in the 70% ethanolic extract.
- Figure S3. CR HPLC-DAD chromatogram of the phenolic compounds presents in the 70% ethanolic extract.
- Figure S4. CB HPLC-DAD chromatogram of the phenolic compounds presents in the 70% ethanolic extract.
- Antioxidants Concentration in Free Radical Scavenging Assay;
- Figure S5. ABTS antioxidants concentration in free radical scavenging assay.
- Figure S6. DPPH antioxidants concentration in free radical scavenging assay.
- Figure S7. H _2_O _2_ antioxidants concentration in free radical scavenging assay.
- Figure S8. HI and TP aqueous extracts antimicrobial activity assay.
- Figure S9. HI and TP 70% ethanolic extracts antimicrobial activity assay.
- Figure S10. CR, CB and EH aqueous extracts antimicrobial activity assay.
- Figure S11. CR, CB and EH 70% ethanolic extracts antimicrobial activity assay.
- Figure S12. Plant identification voucher.

Data are available under the terms of the Creative Commons Zero "No rights reserved" data waiver (CC0 1.0 Public domain dedication).

## References

[1] Kirtikar KR Basu BD Basu LM: Indian Medicinal Plants. Dehradun,1991;181 Reference Source

[2] Burkill HM: The useful plants of West Tropical Africa. Families S–Z, Addenda, Royal Botanic Gardens, Kew Richmond, United Kingdom.2004;5(2):686.

[3] Anonymous: The Wealth of India. CSIR, New Delhi,1985;29–30. Reference Source

[4] Ghosh P Biswas S Dutta A: Evaluation of phytochemical constituents and antioxidant property of leaf acetone extracts of five herbaceous medicinal weeds. *J Pharm Sci & Res.* 2019;11(8):2806–2813. Reference Source

[5] Ghosh P Das P Das C: Morphological characteristics and phyto-pharmacological detailing of Hatishur ( *Heliotropium indicum* Linn.): A concise review. *J Pharmacogn Phytochem.* 2018;7(5):1900–1907. Reference Source

[6] Ghosh P Biswas S Biswas M: Morphological, ethno biological and phytopharmacological attributes of *Tridax procumbens* Linn. (Asteraceae): A review. *International Journal of Scientific Research in Biological Sciences.* 2019;6(2):182–191. 10.26438/ijsrbs/v6i2.182191

[7] Ghosh P Chatterjee S Das P: Natural habitat, phytochemistry and pharmacological properties of a medicinal weed – *Cleome rutidosperma* DC. (Cleomaceae): A comprehensive review. *Int J Pharm Sci Res.* 2019;10(4):1605–1612. 10.13040/IJPSR.0975-8232.10(4).1605-12

[8] Ghosh P Dutta A Biswas M: Phytomorphological, chemical and pharmacological discussions about *Commelina benghalensis* Linn. (Commelinaceae): A review. *Pharma Innov.* 2019;8(6):12–18. Reference Source

[9] Ghosh P Ghosh C Das S: Botanical description, phytochemical constituents and pharmacological properties of *Euphorbia hirta* Linn.: A review. *Int J Health Sci Res.* 2019;9(3):273–286. Reference Source

[10] Wiseman H Halliwell B: Damage to DNA by reactive oxygen and nitrogen species: role in inflammatory disease and progression to cancer. *Biochem J.* 1996;313(Pt 1):17–29. 10.1042/bj3130017 8546679 PMC1216878

[11] Sahoo A Marar T: Phytochemical analysis, antioxidant assay and antimicrobial activity in leaves extracts of *Cerbera odollam Gaertn*. *Pharmacog J.* 2018;10(2):285–92. 10.5530/pj.2018.2.50

[12] Dhanapal V Samuel TB Muddukrishniah K: Screening of Euphorbia Hirta extracts for antioxidant activity. *Indian Journal of Medical Research and Pharmaceutical Sciences.* 2018;5(6):1–15. 10.5281/zenodo.1291909

[13] Malliga E Dhanarajan MS Rajalakshmi A: Analysis of phytochemicals, antibacterial and antioxidant activities of *Moringa oleifera* Lam. leaf extract- An *in vitro* study. *Int J Drug Dev Res.* 2014;6(4):173–180. Reference Source

[14] Vinoth B Manivasagaperumal R Balamurugan S: Phytochemical analysis and antibacterial activity of *Moringa oleifera* LAM. *International Journal of Scientific Research in Biological Sciences.* 2012;2(3):98–102. Reference Source

[15] Sen A Batra A: evaluation of antimicrobial activity of different solvent extracts of medicinal plant: *Melia azedarach* L. *Int J Curr Pharm Res.* 2002;4(2):67–73. Reference Source

[16] Balaji RM Chitra J Sundaram KM: Studies on antidiabetic activity of indian medicinal plants using alpha-amylase and alpha-glucosidase inhibitory activity- A pathway to antidiabetic drugs. *World Journal of Medical Sciences.* 2015;12(3):207–212. Reference Source

[17] Srinivasan, R Dhanalekshmi MU Gowri T: *Terminalia paniculata* bark extract for antidiabetic activity. *Int J Pharm Sci Res.* 2016;7(3):1331–1337. Reference Source

[18] Gulati V Harding IH Palombo EA: Enzyme inhibitory and antioxidant activities of traditional medicinal plants: potential application in the management of hyperglycemia. *BMC Complement Altern Med.* 2012;12: 77. 10.1186/1472-6882-12-77 22713130 PMC3502323

[19] Shilpa K Nimmy C Prerana S: Investigation of anti-arthritic activity ( *in-vitro* models) of *Hibiscus hispidissimus* Griffith. *The Journal of Phytopharmacology.* 2018;7(1):60–65. Reference Source

[20] Singh M Soni P Neeraj U: *In-vitro* anti-arthritic activity of *Manilkara zapota* Linn. *Asian J Pharm Tech.* 2011;1(4):123–124. Reference Source

[21] Sivakumar M Chamundeeswari D Susithra E: Comparative *in-vitro* anti-arthritic studies on the various extracts of *Glycosmis pentaphylla* DC roots. *J Pharm Res.* 2014;8(7):986–989. Reference Source

[22] Srikanth I Puroshotam K Nandeeshwar P: Evaluation of *in vitro* anti-urolithiatic activity of *Ipomea aquatica.* *Int Res J Pharm.* 2018;9(5):8–10. 10.7897/2230-8407.09566

[23] Celestin BR Shijikumar PS Sirajuddin MK: *In-vitro* anti-urolithiatic activity of *Commelina benghalensis* Linn. *Asian J of Research in Chem and Pharmaceutical Sci.* 2017;5(4):150–153. Reference Source

[24] Saravanasingh K Ramamurthy M Parthiban P: *In-vitro* anti-urolithiatic activity of aerial parts of *Aerva lanata* (L.) Juss. *IJCRMS.* 2016;2(3):24–27. Reference Source

[25] Garg A Darokar MP Sundaresan V: Anticancer activity of some medicinal plants from high altitude evergreen elements of Indian Western Ghats. *J Res Educ Indian Med.* 2007;1–6. Reference Source

[26] Singleton VL Orthofer R Lamuela-Raventos RM: Analysis of total phenols and other oxidation substrates and antioxidants by means of Folin-Ciocalteau reagent. *Meth Enzymol.* 1999;299:152–78. 10.1016/S0076-6879(99)99017-1

[27] Zhishen J Mengcheng T Jianming W: The determination of flavonoid contents in mulberry and their scavenging effects on superoxide radicals. *Food Chem.* 1999;64(4):555–559. 10.1016/S0308-8146(98)00102-2

[28] Broadhurst RB Jones WT: Analysis of condensed tannins using acidified vanillin. *J Sci Food Agric.* 1978;29(9):788–794. 10.1002/jsfa.2740290908

[29] Fazel S Hamidreza M Rouhollah G: Spectrophotometric determination of total alkaloids in some Iranian medicinal plants. *Journal of Applied Horticulture.* 2008;32(1):17–20. Reference Source

[30] Solich P Sedliakova V Karlicek R: Spectrophotometric determination of cardiac glycosides by flow-injection analysis. *Analytica Chimica Acta.* 1992;269(2):199–203. 10.1016/0003-2670(92)85403-S

[31] Dey P Dutta S Chaudhuri TK: Phytochemical analysis of the leaves of *Clerodendrum viscossum* Vent. *Int J Pharm Pharm Sci.* 2014;6(2):254–258. Reference Source

[32] Poornima GN Ravishankar RV: Evaluation of phytonutrients and vitamin contents in a wild yam, *Dioscorea belophylla* (Prain) Haines. *Afr J Biotechnol.* 2009;8(6):971–973. 10.4314/ajb.v8i6.59997

[33] Hanfer M Cheriet T Menad A: HPLC profile and *in vitro* antioxidant properties of the *n*-butanol extract of *Linaria tingitana* Boiss. and Reut. *J Chem Pharm Res.* 2019;11(3):50–58. Reference Source

[34] Negro C Aprile A Luvisi A: Phenolic Profile and Antioxidant Activity of Italian Monovarietal Extra Virgin Olive Oils. *Antioxidants.* 2019;8:161. 10.3390/antiox8060161 31195713 PMC6617199

[35] Re R Pellegrini N Proteggente A: Antioxidant activity applying an improved ABTS radical cation decolorization assay. *Free Radic Biol Med.* 1999;26(9–10):1231–37. 10.1016/s0891-5849(98)00315-3 10381194

[36] Tripathi R Mohan H Kamat JP: Modulation of oxidative damage by natural products. *Food Chem.* 2007;100(1):81–90. 10.1016/j.foodchem.2005.09.012

[37] Rajurkar NS Hande SM: Estimation of phytochemical content and antioxidant activity of some selected traditional Indian medicinal plants. *Indian J Pharm Sci.* 2011;73(2):146–151. 10.4103/0250-474x.91574 22303056 PMC3267297

[38] Shen Q Zhang B Xu R: Antioxidant activity *in vitro* of the selenium-contained protein from the Se-enriched *Bifidobacterium animalis* 01. *Anaerobe.* 2010;16(4):380–386. 10.1016/j.anaerobe.2010.06.006 20601030

[39] Thai PK Unaroj B Crosby K: Comparison of ABTS, DPPH, FRAP, and ORAC assays for estimating antioxidant activity from guava fruit extracts. *J Food Compost Anal.* 2006;19(6):669–75. 10.1016/j.jfca.2006.01.003

[40] Ruch RJ Cheng SJ Klaunig JE: Prevention of cytotoxicity and inhibition of intercellular communication by antioxidant catechins isolated from Chinese green tea. *Carcinogenesis.* 1989;10(6):1003–8. 10.1093/carcin/10.6.1003 2470525

[41] Patel A Patel A Patel A: Estmation of flavonoids, polyphenolic content and *in-vitro* anti-oxidant capacity of leaves of *Tephrosia purpurea* Linn. (Leguminosae). *International Journal of Pharma Sciences and Research.* 2010;1(1):66–77.

[42] Minh TN Xuan TD Van TM: Phytochemical Analysis and Potential Biological Activities of Essential Oil from Rice Leaf. *Molecules.* 2019;24(3):546. 10.3390/molecules24030546 30717326 PMC6384862

[43] Mueller L Boehm V: Antioxidant activity of β-carotene compounds in different *in vitro* assays. *Molecules.* 2011;16(2):1055–1069. 10.3390/molecules16021055 21350393 PMC6259600

[44] Esterbauer H Schwarz E Hayn M: A rapid assay for catechol oxidase and lactase using 2-nitro-5-thiobenzoic acid. *Anal Biochem.* 1977;77(2):486–494. 10.1016/0003-2697(77)90262-7 14556

[45] Shanmugapriya A Maneemegalai S: Phytochemical screening, antimicrobial and antioxidant activity of leaf extracts of *Tridax procumbens*. * Int J Res Pharm Chem.* 2017;7(3):320–326. Reference Source

[46] Ghosh, P Biswas M Biswas S: Phytochemical screening, anti-oxidant and anti-microbial activity of leaves of *Cleome rutidosperma* DC. (Cleomaceae). *J Pharm Sci Res.* 2019;11(5):1790–1795. Reference Source

[47] Thorsby E Bratlie A: “A rapid method for preparation of pure lymphocyte suspensions.” In: *Histocompatibility Testing*,Terasaki, P.I., ed.,1970;665–666.

[48] Strober W: Trypan blue exclusion test of cell viability. *Curr Protoc Immunol.* 1997; A3.B.1–A3.B.2. 10.1002/0471142735.ima03bs21 26529666 PMC6716531

[49] Pranabesh G Srabani K Sirshendu C: f1000 Extended data (Phytochemistry and Phytopharmacology of 5 Medicinal Weeds). *figshare Dataset.* 2020 10.6084/m9.figshare.12205631

[50] Pranabesh G Srabani K Sirshendu C: Phytochemical Composition Analysis and Evaluation of *In Vitro* Medicinal Properties and Cytotoxicity of Five Wild Weeds. *figshare Dataset.* 2020 10.6084/m9.figshare.12115827.v2 PMC7331102 32676186

[51] Al-Snafi AE: Pharmacology and therapeutic potential of Euphorbia hirta (Syn: Euphorbia pilulifera )-A review. *IOSR Journal of Pharmacy.* 2017;7(3):07–20. 10.9790/3013-0703010720

[52] Jagadeesan P Prasad DA Pandikumar P: Antioxidant and free radical scavenging activities of common wild greens from Tiruvallur District of Tamil Nadu, India. *Indian J Nat Prod Resour.* 2011;2(2):156–163. Reference Source

[53] Chandha S Dave R: *In vitro* models for antioxidant activity evaluation and some medicinal plants possessing antioxidant properties: An overview. *African J Micro Res.* 2009;3(13):981–996. Reference Source

[54] Fukumoto LR Mazza G: Assessing antioxidant and prooxidant activity of phenolic compounds. *J Agric Food Chem.* 2000;48(8):3597–3604. 10.1021/jf000220w 10956156

[55] Sharififar F Nudeh-dehghn G Mirtajaldini M: Major flavonoids with antioxidant activity from *Teucrium polium* L. *Food Chem.* 2008;112(4):885–888. 10.1016/j.foodchem.2008.06.064

[56] Cowan MM: Plant products as antimicrobial agents. *Clin Microbiol Rev.* 1999;12(4):564–582. 10.1128/CMR.12.4.564 10515903 PMC88925

[57] Kasolo JN Bimenya GS Ojok L: Phytochemicals and uses of Moringa oleifera leaves in Ugandan rural communities. *J Med Plant Res.* 2010;4(9):753–757. Reference Source

[58] Hostettmann K Marston A: Saponins.Cambridge: Cambridge University Press.1995;3 Reference Source

[59] Shamili G Santhi G: Identification and characterization of bioactive compounds of leaves of *Justicia gendarussa* Burm. F. *IJSRBS.* 2019;6(1):145–153. 10.26438/ijsrbs/v6i1.145153

[60] Hazra AK Sur TK Chakraborty B: HPLC analysis of phenolic acids and antioxidant activity of some classical ayurvedic Guggulu formulations. *Int J Res Ayurveda Pharm.* 2018;9(1):112–117. 10.7897/2277-4343.09122

[61] Masek A Chrzescijanska E Latos M: Determination of antioxidant activity of caffeic acid and p coumaric acid by using electrochemical and spectrophotometric assays. *Int J Electrochem Sci.* 2016;11:10644–10658. 10.20964/2016.12.73

[62] Chen C: Sinapic acid and its derivatives as medicine in oxidative stress-induced diseases and aging. *Oxid Med Cell Longev.* 2016; 3571614. 10.1155/2016/3571614 PMC4812465 27069529

[63] Katada S Watanabe T Mizuno T: Effects of chlorogenic acid-enriched and hydroxyhydroquinone-reduced coffee on postprandial fat oxidation and antioxidative capacity in healthy men: a randomized, double-blind, placebo-controlled, crossover trial. *Nutrients.* 2018;10(4):525. 10.3390/nu10040525 29690626 PMC5946310

[64] Subramanian SP Bhuwaneshwari S Prasath GS: Antidiabetic and antioxidant potentials of *Euphorbia hirta* leaves extract studied in streptozotocin-induced experimental diabetes in rats. *Gen Physiol Biophys.* 2011;30(3):278–285. 10.4149/gpb_2011_03_278 21952437

[65] Chen YS Er HM: Antioxidant, anti-proliferative and bronchodilatory activities of *Euphorbia hirta* extracts. *MALAYS J SCI.* 2010;29(1):22–29. 10.22452/mjs.vol29no1.4

[66] Basma AA Zakaria Z Latha LY: Antioxidant activity and phytochemical screening of the methanol extracts of *Euphorbia hirta* L. *Asian Pac J Trop Med.* 2011;4(5):386–390. 10.1016/S1995-7645(11)60109-0 21771682

[67] John RW Lee CY: Recent advances in chemistry of enzymatic browning: An overview. *ACS Symposium Series.* 1995;2–7. 10.1021/bk-1995-0600.ch001

[68] Araji S Grammer TA Escobar MA: Novel roles for the polyphenol oxidase enzyme in secondary metabolism and the regulation of cell death in walnut. *Plant Physiol.* 2014;164(3):1191–1203. 10.1104/pp.113.228593 24449710 PMC3938613

[69] Abubakar EMM: Antibacterial activity of crude extracts of *Euphorbia hirta* against some bacteria associated with enteric infections. *J Med Plants Res.* 2009;3(7):498–505. Reference Source

[70] Kuspradini H Wulandari I Putri AS: Phytochemical, antioxidant and antimicrobial properties of *Litsea angulata* extracts. [version 2; peer review: 3 approved]. *F1000Res.* 2019;7:1839. 10.12688/f1000research.16620.2 30774930 PMC6357992

[71] Perumal S Mahmud R Ramanathan S: Anti-infective potential of caffeic acid and epicatechin 3-gallate isolated from methanol extract of *Euphorbia hirta.* (L.) against *Pseudomonas aeruginosa.* *Nat Prod Res.* 2015;29(18):1766–9. 10.1080/14786419.2014.999242 25571920

[72] Khan SR: Is oxidative stress, a link between nephrolithiasis and obesity, hypertension, diabetes, chronic kidney disease, metabolic syndrome? *Urol Res.* 2012;40(2):95–112. 10.1007/s00240-011-0448-9 22213019 PMC5683185

[73] Kumari S Gupta AK: Antiurolithic activity of *Euphorbia hirta* plant extracts. *Int J Pharm Res.* 2018;9(2):21–24. Reference Source

[74] Kalpana S Rai TS Niramaladevi R: Effect of *Tridax procumbens* extract on calcium oxalate crystallization under *in vitro* conditions. *Adv Appl Sci Res.* 2014;5(3):411–416. Reference Source

[75] Fadhilah K Wahyuono S Astuti P: A bioactive compound isolated from Duku ( *Lansium domesticum* Corr) fruit peels exhibits cytotoxicity against T47D cell line. [version 1; peer review: awaiting peer review]. *F1000Res.* 2020;9:3 10.12688/f1000research.21072.1 PMC8185580 34136135

