# Supplementary material for: Phytochemical composition analysis and evaluation of in vitro medicinal properties and cytotoxicity of five wild weeds: A comparative study
Source: F1000Res. 2020 Jun 2;9:493. [Version 1] doi: 10.12688/f1000research.22966.1 (PMC7331102; doi:10.12688/f1000research.22966.1)
Supplement: Peer review history

# Peer review: Reviewer response for version 1

**DOI:** 10.5256/f1000research.25355.r64766

**Published:** 2020-07-01

**Authors:** Soumya Sasmal

**Affiliations:** 1 Division of Biotechnology, Netaji Subhas University of Technology, New Delhi, Delhi, India

1. Kindly state why the study conducted using only 70% ethanol. What's the reason behind not using 100% or 50% ethanol?
2. Provide the best pictures of Petri plates in the manuscript for a better understanding of the zone of Inhibition study.
3. How do the authors determine the freshness of the leaves? (Size, Color, age of leaves). Kindly state the same in the "Methods" section.

Is the work clearly and accurately presented and does it cite the current literature?

Yes

If applicable, is the statistical analysis and its interpretation appropriate?

Yes

Are all the source data underlying the results available to ensure full reproducibility?

Yes

Is the study design appropriate and is the work technically sound?

Yes

Are the conclusions drawn adequately supported by the results?

Yes

Are sufficient details of methods and analysis provided to allow replication by others?

Yes

Reviewer Expertise:

Biochemical engineering

I confirm that I have read this submission and believe that I have an appropriate level of expertise to confirm that it is of an acceptable scientific standard.

# Peer review: Reviewer response for version 1

**DOI:** 10.5256/f1000research.25355.r64219

**Published:** 2020-06-30

**Authors:** Md Mustafizur Rahman

**Affiliations:** 1 Pharmacy Discipline, Khulna University, Khulna, Bangladesh

The manuscript addressed interesting questions and came up with valuable findings. However, I think that the authors need to address a number of points mentioned below:

**Overall:**

- In the introduction, they should mention the rationale of taking the 4 weeds from the same origin.
- They should justify why they used aqueous and 70% ethanolic media for extraction rather than using any non-polar medium. What difference did they expect from aqueous and 70% ethanolic media?

**Abstract:**

- Although the authors described in methods and results they did not mention anything about *In vitro* antimicrobial activity, anti-diabetic activity, anti-arthritic activity, anti-lithiatic activity findings.

**Methods:**

- Please mention the time and duration of the plant collection. In addition, the authors did not mention how they dried the filtrate after clarification by filtration.
- Collection of microbial strains - Check the last line West 'Bengal and India' where ánd' to be deleted.
- In PPO test **, **no comparison shown between two types of extracts.

**Result:**

- Why did the authors carried out HPLC-DAD analysis for only 70% ethanolic extracts, not for aqueous extract.
- Number of Figures in the study is too high. We suggest the authors combine Figures 8-11 under one heading *In vitro* antioxidant activity.

**Conclusions**:

- Should be more precise and short. It should contain only their own findings not the findings of others which should be discussed under Discussion.

Is the work clearly and accurately presented and does it cite the current literature?

Yes

If applicable, is the statistical analysis and its interpretation appropriate?

Yes

Are all the source data underlying the results available to ensure full reproducibility?

Yes

Is the study design appropriate and is the work technically sound?

Partly

Are the conclusions drawn adequately supported by the results?

Partly

Are sufficient details of methods and analysis provided to allow replication by others?

Partly

Reviewer Expertise:

Medical Pharmacology, Natural product pharmacology, Biopharmaceutics, cancer biology, Pharmacogenetics

I confirm that I have read this submission and believe that I have an appropriate level of expertise to confirm that it is of an acceptable scientific standard, however I have significant reservations, as outlined above.

# Peer review: Reviewer response for version 1

**DOI:** 10.5256/f1000research.25355.r64218

**Published:** 2020-06-22

**Authors:** Mariateresa Cristani

**Affiliations:** 1 Department of Chemical, Biological, Pharmaceutical, and Environmental Sciences, University of Messina, Messina, Italy

The manuscript is provides interesting findings; however, several points need to be addressed:

 About plant material: it is important know the time of the year of harvesting.

 Leaf extract preparation: a the beginning you indicate '1 g of powdered leaf was extracted with 50 ml solvent'

 In Leaf extract preparation ' 100 mg dry weights of each plant leaves were mixed, vortexed in 1 ml of solvent; explain it.

 About saponins test it is not clear why you report just one kind of result in figure 6, if you obtain it from the leaf powder and not from the 2 different extracts, I cannot understand why you make correlation with the two different kind of extracts.

 The same for the ppo test. You did not compare the 2 different extracts.

 About the figure 7 HPLC is not clear why you left numbers and names, it is really difficult read it.

 Please, could you add numbers for each compound in the figure 7 and the same numbers and Rt  in the table 1.

 Moreover, you report  'quantification of different bioactive compounds present in the extracts' .

 Why have you identified compounds by the HPLC-DAD for ethanolic extract only.

 Table 2: Zone of inhibitions of leaves extracts against different bacterial strains.

  Please, write 0 or Nil

Is the work clearly and accurately presented and does it cite the current literature?

Partly

If applicable, is the statistical analysis and its interpretation appropriate?

Yes

Are all the source data underlying the results available to ensure full reproducibility?

Partly

Is the study design appropriate and is the work technically sound?

Partly

Are the conclusions drawn adequately supported by the results?

Partly

Are sufficient details of methods and analysis provided to allow replication by others?

Yes

Reviewer Expertise:

Pharmacology, Pharmacognosy and Toxicology

I confirm that I have read this submission and believe that I have an appropriate level of expertise to confirm that it is of an acceptable scientific standard, however I have significant reservations, as outlined above.
